# Hybrid deep modeling of a CHO-K1 fed-batch process: combining first-principles with deep neural networks

**DOI:** 10.3389/fbioe.2023.1237963

**Published:** 2023-09-08

**Authors:** José Pinto, João R. C. Ramos, Rafael S. Costa, Sergio Rossell, Patrick Dumas, Rui Oliveira

**Affiliations:** ^1^ LAQV-REQUIMTE, Department of Chemistry, NOVA School of Science and Technology, NOVA University Lisbon, Caparica, Portugal; ^2^ GlaxoSmithKline, Rixensart, Belgium

**Keywords:** hybrid modeling, deep neural networks, first-principles, ADAM, stochastic regularization, CHO-K1 cells, biopharma 4.0

## Abstract

**Introduction:** Hybrid modeling combining First-Principles with machine learning is becoming a pivotal methodology for Biopharma 4.0 enactment. Chinese Hamster Ovary (CHO) cells, being the workhorse for industrial glycoproteins production, have been the object of several hybrid modeling studies. Most previous studies pursued a shallow hybrid modeling approach based on three-layered Feedforward Neural Networks (FFNNs) combined with macroscopic material balance equations. Only recently, the hybrid modeling field is incorporating deep learning into its framework with significant gains in descriptive and predictive power.

**Methods:** This study compares, for the first time, deep and shallow hybrid modeling in a CHO process development context. Data of 24 fed-batch cultivations of a CHO-K1 cell line expressing a target glycoprotein, comprising 30 measured state variables over time, were used to compare both methodologies. Hybrid models with varying FFNN depths (3-5 layers) were systematically compared using two training methodologies. The classical training is based on the Levenberg-Marquardt algorithm, indirect sensitivity equations and cross-validation. The deep learning is based on the Adaptive Moment Estimation Method (ADAM), stochastic regularization and semidirect sensitivity equations.

**Results and conclusion:** The results point to a systematic generalization improvement of deep hybrid models over shallow hybrid models. Overall, the training and testing errors decreased by 14.0% and 23.6% respectively when applying the deep methodology. The Central Processing Unit (CPU) time for training the deep hybrid model increased by 31.6% mainly due to the higher FFNN complexity. The final deep hybrid model is shown to predict the dynamics of the 30 state variables within the error bounds in every test experiment. Notably, the deep hybrid model could predict the metabolic shifts in key metabolites (e.g., lactate, ammonium, glutamine and glutamate) in the test experiments. We expect deep hybrid modeling to accelerate the deployment of high-fidelity digital twins in the biopharma sector in the near future.

## 1 Introduction

Chinese hamster ovary (CHO) cells are the most widely used host system for the industrial production of biologics. They cover more than 70% of the mammalian cell-based therapeutic proteins production (Vcelar et al., 2018). They present several advantages such as well-established large-scale cultivation with high productivity (cell densities higher than 20 Mcell/mL with protein titer as high as 10 g/L), human-like N-glycosylation, well-established molecular biology techniques and an impressive track record of approvals by the U.S. Food and Drug Administration (FDA) (Galleguillos et al., 2017). Given its industrial relevance, many companies have established CHO-cell platforms to streamline process development of many different molecule candidates in a short timeframe (e.g., (Mora et al., 2018)). Different upstream tasks such as clone screening, culture media customization and reactor optimization should be integrated in a rational way to improve the efficiency of process development. The adoption of high-throughput screening technologies allied with advanced digitalization tools for data analysis, mathematical modeling and control across the different development stages are key factors to improve process development efficiency (Hole et al., 2021).

There are currently three main mathematical modeling formalisms that are used for the digitalization of biopharmaceutical processes: First-Principles or mechanistic modeling (e.g., Hartmann et al., 2022; Monteiro et al., 2023) data-based or machine learning (ML) (e.g., Mowbray et al., 2022; Mowbray et al., 2022; Helleckes et al., 2023) and hybrid mechanistic/ML (e.g., Badr and Sugiyama, 2020; Bayer et al., 2023; Narayanan et al., 2023). Mechanistic modeling relies on prior process knowledge and requires less process data. They are more complex to develop but tend to extrapolate better outside the domain of experience. The intrinsic complexity of biological systems is however a critical limitation for the deployment of mechanistic models in an industrial context (Badr et al., 2021). Conversely, ML relies almost exclusively on process data with minimal prior knowledge requirements. Artificial neural networks (ANNs) are currently the most popular ML technique in bioprocess engineering, followed by ensemble learning, multivariate data analysis, support vector machines and gaussian processes (Mowbray et al., 2022). As key advantage, ANNs were shown to be universal nonlinear function approximators (Cybenko, 1987). Due to the typically large number of parameters and unstructured nature, ANNs require large data sets for training and are prone to overfitting and poor generalization (Bejani and Ghatee, 2021). ANNs and ML methods in general are easier to develop but require large amounts of data that are costly, time-consuming and difficult to reuse. ML models tend to describe better inside the domain of experience (e.g., better interpolation) but are less reliable at extrapolating in comparison to mechanistic models. Hybrid models combine mechanistic and ML techniques in a common workflow and share the pros and cons of both techniques (e.g., Psichogios and Ungar, 1992; Oliveira, 2004; Teixeira et al., 2005; Teixeira et al., 2007; von Stosch et al., 2014; Kurz et al., 2022; Pinto et al., 2019). The mechanistic modules allow to decrease the complexity of the ML modules within the hybrid model and as such the overall data requirements are decreased. Moreover, the ML modules fill the gaps of the mechanistic modules for which knowledge is still lacking. Narayanan et al. (2022) studied the impact of increasing the amount of prior knowledge (e.g., material balances, reaction stoichiometry and reaction kinetics) in the hybrid model of a cell culture process. Between a fully data-driven (or ML model) and a fully mechanistic model, there are different degrees of hybridization possible depending on the amount of prior knowledge included in the hybrid model. The authors concluded that the inclusion of unbiased prior knowledge progressively improves the performance of the hybrid model. Unsurprisingly, fully data-driven models showed poor performance particularly when data is scarce. Rogers et al. (2023) have also investigated the optimal amount of prior knowledge to incorporate in a hybrid bioprocess model. The authors concluded that the inclusion of correct kinetic information generally improves the performance of the hybrid model. The inclusion of incorrect kinetic assumptions may however create inductive bias that decreases the performance of the hybrid model. Due to the flexible trade-off between prior knowledge and data availability, hybrid modeling is becoming a method of choice to develop digital twins in the realm of Biopharma 4.0 (e.g., Badr and Sugiyama, 2020; Yang et al., 2019; Sansana et al., 2021; Sokolov et al., 2021; Badr and Sugiyama, 2020; Narayanan et al., 2023, Bayer et al., 2022, Bayer et al. 2023).

Being the preferred host system in biopharma, CHO cultivation processes have been the object of several hybrid modeling studies (Table 1). Most of previous studies combined macroscopic material balance equations of extracellular species with some machine learning/statistical modeling methods with predominance of shallow FFNNs with a single hidden layer. The macroscopic material balance equations are translated to systems of Ordinary Differential Equations (ODEs) describing bioreactor dynamics. The machine learning component is typically dedicated to model biological kinetics, which are parts of the system lacking mechanistic basis. The number of biochemical species has been limited to 2–12 species. Typically, the viable cell count, concentrations of the target molecule and the concentrations of key central carbon metabolites such as glucose, lactate, glutamine, glutamate and ammonium. A recent study by Doyle et al. (2023) has also covered amino acids dynamics. The training method is either coupled or uncoupled. In the latter case, the machine learning component is isolated from the mechanistic model and trained as a standalone module. In the former case, the mechanistic and machine learning models are parametrized in a common mathematical structure and trained together. Uncoupled training has been adopted by Kotidis et al. (2021) to develop a hybrid model of glycosylation critical quality attributes in CHO cultures. The N-linked glycosylation was described by a FFNN with 2 hidden layers, while the cell growth and metabolism were described by a mechanistic model based on a system of Differential and Algebraic Equations (DAEs) (Kotidis et al., 2019). The FFNN was trained as a standalone model on data generated by the mechanistic model using the *TensorFlow* package in Python 3.7. The final trained FFNN and the mechanistic model were assembled in a hybrid workflow in gPROMS v.5.0.1. Coupled training has been the preferred approach for material balance + FFNN hybrid models, following the scheme originally proposed by Psichogios and Ungar (1992). The sum of square error between measured and calculated concentrations is minimized during the training using the Levenberg-Marquardt (LMM) algorithm. Since the FFNN outputs cannot be directly compared with measured properties, this method is termed indirect training. The indirect sensitivity equations are employed to compute the gradients of measured concentrations in relation to neural network weights (Psichogios and Ungar, 1992; Oliveira, 2004). Cross-validation techniques are employed to avoid overfitting. Following the coupled training approach with cross-validation, Bayer et al., 2022 compared fully mechanistic and shallow hybrid modeling for characterization of a CHO cultivation process. The authors concluded that the prediction accuracy of the shallow hybrid model was always superior to the mechanistic model irrespective of the utilized data partition. Due to its’ higher fitting power, the shallow hybrid model prediction accuracy was more sensitivity to data resampling than the fully mechanistic model. Every hybrid model in Table 1 is of dynamic nature except the one by Ramos et al. (2022). The authors have used a large genome-scale network with 788 reactions as mechanistic component combined with a Principal Component Analysis (PCA) model. The overall hybrid model is of static nature, solved by linear programming under the pseudo steady-state hypothesis, i.e., by hybrid Flux Balance Analysis (hybrid FBA).

**TABLE 1:** Compilation of CHO hybrid modeling studies.

First-principles	Machine learning	Training method	Cross validation	Objective	References
Macroscopic material balances (2 species)	Shallow FFNN (tanh hidden nodes)	Levenberg-Marquardt; coupled	Yes	Prediction of culture dynamics; Quality-By-Design	Bayer et al. (2021)
Macroscopic material balances (7 species)	Shallow FFNN (tanh hidden nodes)	Levenberg-Marquardt; coupled	Yes	Prediction of culture dynamics; Quality-By-Design	Bayer et al. (2022), Bayer et al. (2023)
Macroscopic material balances (4 species)	Shallow FFNN (tanh hidden nodes)	Levenberg-Marquardt; coupled	Yes	Optimize viable cell density	Nold et al. (2023)
Macroscopic material balances (4 species)	Shallow FFNN (tanh hidden nodes)	MATLAB *fminunc* function; coupled	Yes	Prediction of culture dynamics; Quality-By-Design	Narayanan et al. (2019)
Macroscopic material balances (6 species)	Gaussian Process regression	Maximum likelihood estimator; uncoupled	Yes	Prediction of culture dynamics across different products	Hutter et al. (2021)
Mechanistic kinetic models (12 species)	Deep FFNN with 2 hidden layers (softmax/sigmoid hidden nodes)	Python 3.7 Tensorflow/gPROMS v.5.0.1; uncoupled	Yes	Prediction of culture dynamics and mAb glycosylation	Kotidis et al. (2021)
Macroscopic material balances (5 species)	Set of Shallow FFNN (tanh hidden nodes)	Levenberg-Marquardt; uncoupled	Yes	Software sensor of r-tPA production	Senger and Karim (2003)
Macroscopic material balances (4 species)	Principle Component Regression (PCR)	PCA + least squares regression; uncoupled	Yes	Prediction of culture dynamics	Okamura et al. (2022)
Macroscopic material balances (24 species)	Saturation and sigmoidal functions	Least squares regression; uncoupled		Automated assembly of dynamic model	Doyle et al. (2023)
CHO-K1 Genome-scale network (788 reactions; 686 species)	PCA of reaction rates of extracellular species	Linear programming; coupled	Yes	Hybrid FBA; Culture media design	Ramos et al. (2022)

Most previous hybrid modeling studies have combined material balance equations with shallow FFNNs or other nondeep machine learning techniques. In the field of neural networks, Deep neural networks have however been shown to have a general advantage over their shallow counterparts thanks to their ability to approximate more complex functions with a lower number of parameters and being less prone to overfitting (Delalleau and Bengio, 2011; Eldan and Shamir, 2016; Mhaskar and Poggio, 2016; Liang and Srikant, 2017). Training of deep structures also requires special care, with the ADAM method (Kingma, 2014) being commonly used due to its robustness and lower sensitivity to local optima. Along with the training approach, the use of stochastic regularization techniques has also shown to be very effective at avoiding overfitting (Hinton et al., 2012; Srivastava et al., 2014; Koutsoukas et al., 2017).

Only very recently, hybrid modeling is incorporating deep neural networks and deep learning into its framework (Bangi and Kwon, 2020; Pinto et al., 2022; Bangi and Kwon, 2023). Pinto et al. (2022) investigated the use of ADAM and stochastic regularization in a hybrid modeling context concluding that the predictive power of deep hybrid models was significantly improved. None of these techniques have been applied to CHO processes (Table 1). In this study, we thus investigate deep learning techniques based on ADAM and stochastic regularization in a hybrid modeling context with application to a CHO-K1 fed-batch process. The deep learning method is compared with the classical shallow method based on the LMM algorithm, indirect sensitivity equations and cross-validation. The rest of this paper is organized as follows: in Section 2 we introduce the methodology. Section 3 contains the results for the case study, and a discussion of the results in section 4. Section “Conclusion” gives the final remarks and sums up the main findings.

## 2 Methods

### 2.1 CHO-K1 experimental dataset

Data from 24 fed-batch reactor experiments with a CHO-K1 cell line coding for a target glycoprotein were used to compare the hybrid modeling methodologies. Briefly, the cells were pre-cultured in shake-flasks (Corning, NY, United States) at 37°C in a proprietary chemically defined medium. The inoculum was transferred to 250 mL stirred microcarrier vessel (Ambr^®^ 250 workstation, Sartorius, Göttingen, Germany) for antigen production. Stirring was kept at around 20 W/m³. Dissolved oxygen was controlled at 30% of saturation by sparging pure oxygen. The pH was controlled at 7.0 with a 0.5 M NaOH solution and CO_2_ sparging. The reactors were seeded at 3.0 Mcell/mL. They followed a batch/fed-batch phase for viable cells expansion. Once a threshold viable cell density was reached, the temperature was decreased to 33°C to induce antigen production. The antigen production phase was carried out in fed-batch mode with varying feeding compositions of amino acids, glucose and pyruvate. The whole process lasted approximately 12 days. Samples were taken daily. Viable cell density and viability were assayed using a Vi-Cell cell counter (Beckman, Indianapolis, United States). Glucose, lactate, pyruvate, glutamine, ammonium, glycerol and lactate dehydrogenase were assayed using a CedexBio-HT metabolite analyzer (Roche, Penzberg, Germany). The antigen quantification was performed off-line with an Octet HTX (Pall, NY, United States). The remaining metabolites and amino acids were assayed off-line by Nuclear Magnetic Resonance spectroscopy at Eurofins Spinnovation (Oss, Netherlands). A total of 30 concentrations were measured at each time point (with few exceptions): viable cell count (Xv), glycoprotein (P), glucose (Glc), lactate (Lac), glutamine (Gln), glutamate (Glu), ammonium (NH4), pyruvate (Pyr), glycerol (Glyc), citrate (Cit), alanine (Ala), arginine (Arg), asparagine (Asn), aspartate (Asp), L-cystine (Lcystin), glycine (Gly), histidine (His), isoleucine (Ile), leucine (Leu), lysine (Lys), methionine (Met), phenylalanine (Phe), proline (Pro), serine (Ser), threonine (Thr), tryptophane (Trp), tyrosine (Tyr), valine (Val), acetate (Ac) and formate (For). The data were assumed to be corrupted by heterogenous gaussian noise. The measurement error standard deviations were assumed to be of 5% for P, 10% for Xv and 20% for remaining metabolites, based on equipment calibration data. The data reliability was pre-assessed by statistical analysis of metabolic fluxes in the exponential growth and production phases. The spread of data was analyzed in a boxplot of metabolic fluxes. No outlying reactor experiments were identified. All the 24 reactor experiments were used for modeling thus none discarded due to reliability issues. More details regarding the experimental protocol and data pre-assessment are provided by Ramos et al. (2022).

### 2.2 CHO-K1 synthetic dataset

In addition to the experimental dataset, a synthetic dataset was created based on the metabolic model proposed by Robitaille et al. (2015). A synthetic dataset is useful in this context to better assess the ability of the hybrid modeling methods to describe the intrinsic process behavior irrespective of measurement noise. Simulations of this model were performed by varying two parameters, namely, the pre-induction feeding rate and the post-induction feeding rate. A central composite design of experiments (CC-DoE) was applied to obtain 9 combinations of the two feed rates. This resulted in 9 fed-batch simulated experiments. The dynamic model has 21 intracellular species and 25 extracellular species. The intracellular species were hidden to the hybrid model development. The concentrations of extracellular species were recorded as time series for 240 h with 24 h sampling time and included the following variables: Xv, monoclonal antibody concentration (mAb), Ala, Arg, Asn, Asp, Cysteine (Cys), Glc, Gln, Glu, Pyr, Gly, His, Ile, Lac, Leu, Lys, Met, NH4, Phe, Pro, Ser, Thr, Tyr and Val. The recorded variables from the synthetic dataset were the same as in the experimental dataset, except that Pyr, Glyc, Cit and Ac are not considered in the Robitaille et al (2015) model. Moreover, the target products are different and Robitaille et al (2015) considers cysteine instead of Cystine. Gaussian white noise with standard deviation of 10% of maximum concentration values was added to concentrations time points to mimic (heterogeneous) gaussian measurement error. This synthetic dataset is provided as Supplementary Material.

### 2.3 CHO-K1 hybrid model

**FIGURE 1:**
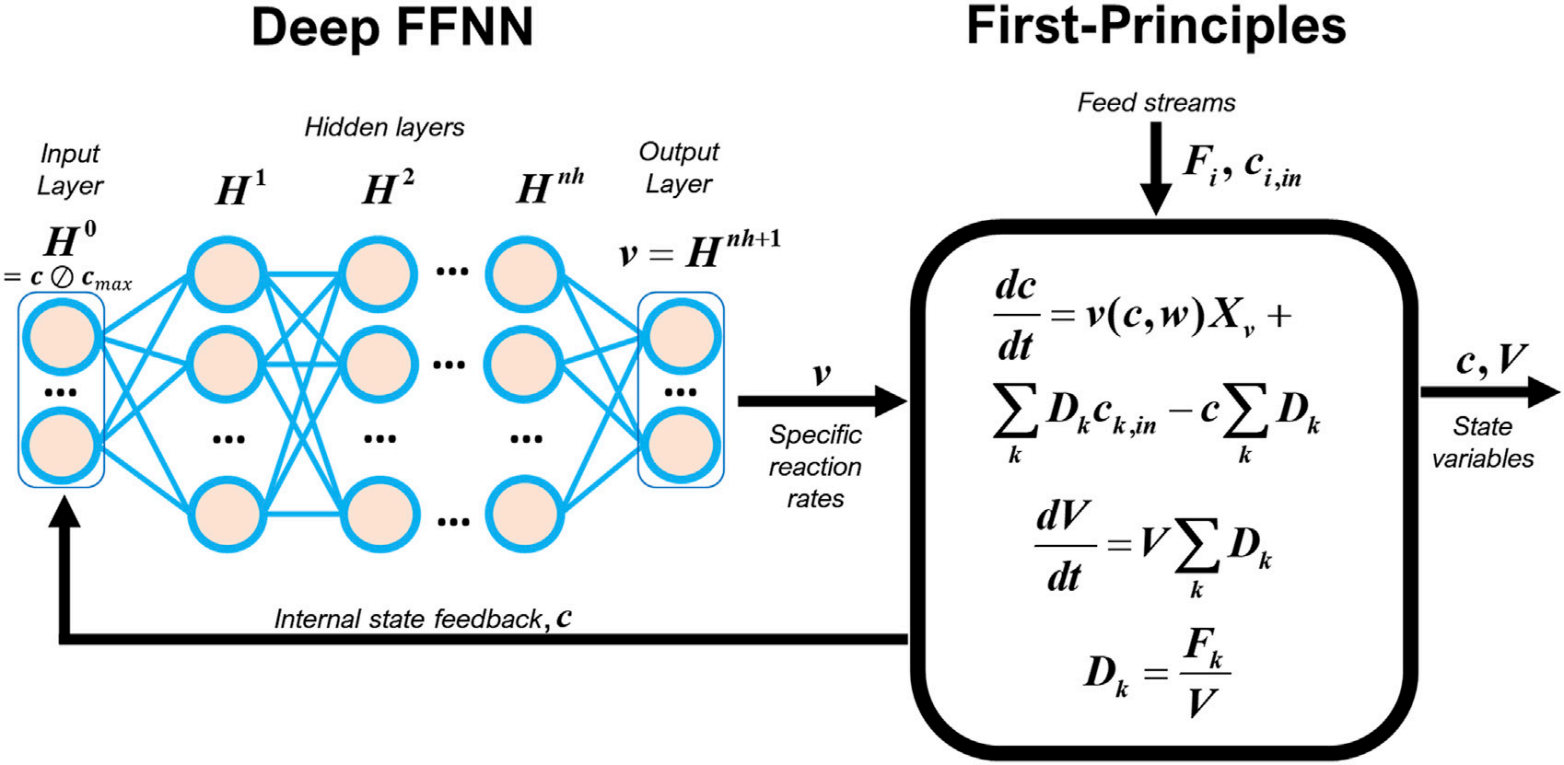
Hybrid model structure of a CHO-K1 fed-batch process.

A standard hybrid model configuration was adopted in this study consisting of a multilayered FFNN connected in series with macroscopic material balance equations (Figure 1). This configuration is similar to previously published studies (Table 1) except for the depth of the FFNN and the training methods employed. The FFNN is dedicated to completely model the reaction kinetics. The dynamics of state variables are modeled by a system of ODEs based on macroscopic material balance equations (First-Principles). Considering a perfectly mixed fed-batch bioreactor with multiple feed streams, the macroscopic material balance equations take the following state-space form:

$$\frac{dc}{dt}=v\left(c,w\right)X_{v}+\sum_{k}D_{k}c_{k,in}-c\sum_{k}D_{k}$$ (1a)

$$\frac{dV}{dt}=V\sum_{k}D_{k}$$ (1b)

$$D_{k}=\frac{F_{k}}{V}$$ (1c)

with t the independent variable time, c the state vector with the concentrations of 30 species (Xv, P, Glc, Lac, Gln, Glu, Nh4, Pyr, Glyc, Cit, Ala, Arg, Asn, Asp, Lcystin, Gly, His, Ile, Leu, Lys, Met, Phe, Pro, Ser, Thr, Trp, Tyr, Val, Ac, For), $v\left(∙\right)$ the specific reaction rates vector of the 30 species, $D=\sum_{k}D_{k}$ the reactor dilution rate (scalar), V the cultivation volume (scalar), $F_{k}$ the feed rate of stream k (there are in total 5 feed streams) and $c_{k,in}$ the vector of species concentrations in feed stream k . The specific reactions rates, $v\left(c,w\right)$ lack mechanistic basis and were thus modeled by a deep FFNN with *nh* hidden layers:

$$H^{0}=c⊘c_{\max}$$ (2a)

$$H^{i}=\sigma \;\left(w^{i}∙H^{i-1}+b^{i}\right),i=1,\ldots ,nh$$ (2b)

$$v=w^{nh+1}∙H^{nh}+b^{nh+1}$$ (2c)

The input layer i=0 (Eq. 2a) with 30 nodes receives the information of normalized concentrations ( $c_{\max}$ is the absolute maximum concentration of the 30 species (vector) and ⊘ the Hadamard division). Each hidden layer i computes a vector of outputs, $H^{i},$ from a vector of inputs, $H^{i-1}$ , which are the outputs of the preceding layer (Eq. 2b). The transfer function of hidden nodes, $\sigma \left(∙\right)$ , was either the hyperbolic tangent function, *tanh,* or the rectified linear unit, *ReLU*. The output layer (Eq. 2c) computed the specific reaction rates vector of the 30 species. The parameters $w=\left\{w^{1},w^{2},\ldots ,w^{nh+1}\right\}$ are the nodes connection weights between layers and $b=\left\{{b^{1},b}^{2},\ldots ,b^{nh+1}\right\}$ the bias weights that need to be optimized data during the training process. The deep hybrid model Eqs 1, 2 were integrated numerically using a Runge-Kutta 4^th^ order ODE solver (in-house developed in MATLAB).

#### 2.3.1 Shallow hybrid modeling method

This study compares shallow and deep hybrid modeling. The shallow structures are represented by Eqs 1, 2 with FFNNs with a single hidden layer and with hyperbolic tangent activation function, *tanh*. Sigmoidal activation functions, and particularly *tanh*, are generally accepted as a default in shallow FFNNs. Many practical studies have corroborated the universal function approximation property derived by Cybenko, (1989). This FFNN architecture has also been the preferred choice in a hybrid modeling context (e.g., Table 1). The training of shallow hybrid models is based on the LMM optimization with the indirect sensitivity equations (to compute gradients) and cross-validation (as early stop criteria). Briefly, the data were partitioned in a training/validation subset (for parameter estimation) and a testing subset (to assess the predictive power). Partitioning was performed batch wise with the amount of data allocated in each partition depending on the context (further details in the results section). The LMM algorithm (*fminunc* function in MATLAB) was adopted to optimize the network parameters, $\left\{w,b\right\}$ , by unconstrained weighted least squares computed on the training data subset only. The inverse of measurement error variance was used as weighting factor in the weighted least squares minimization in order to effectively filter heterogeneous gaussian error (Eq. 3). The objective function gradients were computed by the indirect sensitivity equations following the method described by Oliveira (2004) (more information in the Supplementary Material). Cross-validation was adopted as a stop criterion to avoid overfitting, i.e., the training is stopped when the validation error increases. A data augmentation strategy was used to automatically create the validation data subset from the training subset by adding gaussian noise to the concentrations (Bejani and Ghatee, 2021). The standard deviation of the added noise was the same as the standard deviation of the measured concentration error. This strategy has proven to effectively avoid overfitting to the experimental noise and to produce good generalization models when the data information content is well distributed among the training and testing data subsets (Pinto et al., 2022). For each shallow hybrid structure, the training was repeated 10 times with random weights initialization from the uniform distribution. Only the best result (lowest training/validation error) was kept. Further details are provided as Supplementary Material.

#### 2.3.2 Deep hybrid modeling method

The shallow hybrid models were systematically compared with deep hybrid models. The deep hybrid models are represented by Eqs 1, 2 with FFNNs with multiple hidden layers ( nh≥2 ) and with rectified linear unit (ReLU) hidden nodes. The *tanh* was replaced by the *ReLU* because the latter is generally accepted as a default for several deep neural network architectures including deep FFNNs (Goodfellow et al., 2006). The *ReLU* function solved two main problems associated with the *tanh* function, namely, signal saturation and the vanishing gradients problem that occurs during error backpropagation in networks with multiple hidden layers (Glorot and Bengio, 2010). Instead of the LMM algorithm, deep hybrid models were trained with the ADAM algorithm (in-house implementation). The ADAM algorithm is generally accepted as an efficient method to train deep FFNNs (Kingma, 2014). The use of ADAM in a hybrid modeling context has been recently investigated by Pinto et al. (2022). Briefly, the data were portioned in a training and in a testing subset as for shallow hybrid modeling. The ADAM was adopted to optimize the network parameter, {**w**, **b**}, also in a weighted least squares sense in order to effectively filter heterogeneous gaussian error. The objective function gradients were computed by the semidirect sensitivity equations. The semidirect sensitivity equations method was shown to reduce the training CPU time in comparison to the indirect sensitivity equations method used in shallow hybrid modeling (Pinto et al., 2022). Stochastic regularization with minibatch size (0–1) and weights dropout probability (0–1) was applied to avoid overfitting in replacement of cross-validation normally applied in shallow hybrid modeling. The ADAM with stochastic regularization was run for a sufficiently large number of iterations with the final deep FFNN weights taken at the iteration with minimum training error. The training was performed only once because ADAM is less sensitive to weights initialization. This methodology has been thoroughly investigated by Pinto et al. (2022). Further details are provided as Supplementary Material.

#### 2.3.3 Model performance, selection and implementation

The performances of shallow and deep hybrid models were assessed by the Weighted Mean Square Error (WMSE) computed as follows:

$$WMSE=\frac{1}{T}\;\sum_{t=1}^{T}\frac{{\left.{\left(c\right.}_{t}^{*}-c_{t}\right)}^{2}}{\sigma_{t}^{2}}$$ (3)

with T the number of data examples, $c_{t}^{*}$ the measured concentration at time t , $c_{t}$ the model calculated concentration at time t and $\sigma_{t}$ the standard deviation of measurement at time t . The WMSE was computed separately for the training and testing data subsets. In the case of the synthetic dataset, the test WMSE was computed using $c_{t}^{*}$ with experimental noise (noisy test WMSE) and without noise (noise-free test WMSE).

Model selection was performed by a probabilistic method and by a resampling method. The probabilistic method consisted in the Akaike’s Information Criterion (AIC) with second order bias correction (AICc). The second order correction is needed for small data samples (*T* < 40), eventually converging to the AIC value for very larger samples (Banks and Joyner, 2017). It is computed on the training data subset as follows:

$$AICc=T\;\ln\left(WMSE\right)+2\;nw+\frac{2\;nw\;\left(nw+1\right)}{T-nw-1}$$ (4)

The AICc was adopted to discriminate parsimonious hybrid structures by taking into account the model complexity (i.e., the total number of network parameters, nw ). The model with lowest AICc score was selected as the best model.

Model selection was also performed by a resampling technique. Ten different training and testing data partitions were created by random selection (from the uniform distribution) of reactor experiments allocated either for training or for testing. The training was repeated for every data partition resulting in 10 different models. The respective training and testing WMSE statistics were evaluated. The best model was selected to be the one with the lowest mean test WMSE.

The AICc and the resampling method often led to different model selection conclusions (further discussed in the results section). It is generally accepted that resampling methods are preferred over probabilistic methods for statistical model selection (Tashman, 2000). Therefore, the resampling method, based on the lowest test WMSE, was taken as the final decision metric for the selection of hybrid models.

All the code of shallow and deep hybrid modeling was developed in-house and implemented in MATLAB on a computer with Intel(R) Core(TM) i5-8265U CPU @ 1.60 GHz 1.80 GHz, and 24 GB of RAM. CPU time of the different tests performed were computed as the difference between the result of the “*cputime*” MATLAB function.

## 3 Results

### 3.1 Shallow hybrid modeling of the CHO-K1 synthetic dataset

Shallow hybrid models with varying number of nodes in a single hidden layer with *tanh* activation function were investigated. At this stage, the synthetic dataset was adopted since it allows a better control of the information content distribution among the training and testing data subsets. The training partition was composed of 5 batches with 2,400 training examples (the number of training examples was always higher than the number of FFNN weights). The testing partition was composed of 4 batches with 1920 testing examples. The training experiments were the center and square points of the CC-DoE, whereas the test experiments were the star points of the CC-DoE. The comparatively large testing data subset, generated at the extreme star points of the CC-DoE, represents a challenging extrapolating test for the trained hybrid models. Given the very clear testing rationale, the resampling repetitions were not applied in this case, which allowed to save some CPU time. The training and testing data subsets were always the same with models compared based on the AICc score and on the final test WMSE. The number of nodes of the hidden layer varied between 1 and 15 corresponding to a number of weights between 77 and 805. The training algorithm was the LMM with gradients computed by the indirect sensitivity method. For each structure, the training was repeated 10 times with different weights initialization (classical method). The overall results are shown in Table 2. These results confirm that the number of nodes in the hidden layers has a significant effect on the model performance. The AICc score and the test WMSE did not converge to a common conclusion (discussed below). The shallow structure with lowest AICc had 5 hidden nodes only, which did not correspond to the lowest test error. The shallow structure with highest predictive power had 12 hidden nodes with the lowest noisy and noise-free test WMSE (2.04 and 2.06, respectively). The noisy test WMSE was 32.5% higher than the train WMSE denoting some degree of overfitting of the training data. The AICc criterion miss selected the model with the highest predictive power in this case.

**TABLE 2:** Shallow hybrid modeling results on the CHO-K1 synthetic dataset. Hybrid models had a FFNN with a single hidden layer with hyperbolic tangent activation function and a number of nodes between 1 and 15. The training algorithm was the Levenberg-Marquardt with gradients computed by the indirect sensitivity equations with 1,000 iterations and cross-validation as stop criterion. Training was repeated 10 times for each structure with random weights initialization from the uniform distribution between −0.01 and 0.01 and only the best result was kept. The WMSE-train was computed on the training dataset with 10% gaussian noise in concentrations. WMSE-test (noisy) was computed on the test dataset with 10% gaussian noise in concentrations. WMSE-test (noise free) was computed on the test dataset without noise in the concentrations. The AICc was computed on the same dataset as WMSE-train.

Number of hidden nodes	WMSE -train	WMSE-test (noisy)	WMSE-test (noise free)	AICc	CPU time (hh:mm:ss)	Number of weights
1	6.07	7.46	8.16	4,890	00:13:20	77
2	2.17	3.82	4.32	2,310	00:25:31	129
3	1.81	3.25	3.64	1950	00:30:04	181
4	1.76	2.79	3.15	2000	00:26:44	233
**5**	**1.28**	**4.57**	**4.31**	**1,290**	**00:23:06**	**285**
6	1.52	2.31	2.76	1890	00:27:34	337
7	1.55	2.10	2.18	2070	00:24:58	389
8	1.66	3.09	3.45	2,400	00:30:18	441
9	1.73	2.71	2.79	2,500	00:26:40	493
10	1.60	2.47	2.63	2,450	00:32:20	545
11	1.70	2.73	3.12	2,930	00:28:15	597
**12**	**1.54**	**2.04**	**2.06**	**2,850**	**00:24:52**	**649**
13	1.64	2.70	2.84	3,210	00:32:30	701
14	1.73	6.33	7.14	3,550	00:18:15	753
15	1.54	2.65	2.86	3,460	00:22:18	805

The bold values indicate the optimal model configuration.

### 3.2 Deep hybrid modeling of the CHO-K1 synthetic dataset

Deep hybrid modeling with FFNNs with 2 or 3 hidden layers was investigated on the same synthetic dataset. Models with more than 3 hidden layers did not produce further improvements (results not shown). The activation function in the hidden layer was the *ReLU* in all cases. The training algorithm was the ADAM with standard hyperparameters (Kingma, 2014). Stochastic regularization with optimal minibatch size of 0.8 and weights dropout of 0.2 was adopted, based on a previous study by Pinto et al. (2022). Stochastic regularization coupled with ADAM was shown to be very robust to weights initialization (Pinto et al., 2022) thus the training was carried out only once with a single random weights initialization (between −0.01 and 0.01). The overall results are shown in Table 3. As expected, the complexity of the FFNN has a significant effect on the model performance. The number of weights varied between 315 and 1905, always lower than the number of training examples (2,400). The hybrid structure 10 × 10 × 10 with 765 weights clearly stands out as the best performing structure. The obtained training and testing errors are comparable denoting a successful training without overfitting. Moreover, the noise free test error is clearly below the noisy test error, showing that this model was able to filter noise in the test partition. The AICc of the 10 × 10 × 10 structure was also the lowest among the deep hybrid structures investigated. The AICc and the test WMSE pointed to the same conclusion in this case.

**TABLE 3:** Deep hybrid modeling results on the synthetic CHO-K1 dataset. Hybrid models had a FFNN with 2 or 3 hidden layers with *ReLU* activation function. The training algorithm was the ADAM algorithm run for 1,000 iterations with hyperparameters α=0.001 , β1=0.9 , β2=0.999 and $\eta =1e^{-7}$ . Gradients were computed by the semidirect sensitivity equations. Stochastic regularization was applied with weights dropout of 0.2 and minibatch size of 0.8. The training was repeated only once with random weights initialization from the uniform distribution between −0.01 and 0.01. The WMSE-train was computed on the training dataset with 10% gaussian noise in concentrations. WMSE-test (noisy) was computed on the test dataset with 10% gaussian noise in concentrations. WMSE-test (noise free) was computed on the test dataset without noise in the concentrations. The AICc was computed on the same dataset as WMSE-train.

Number of hidden nodes	WMSE train	WMSE test (noisy)	WMSE test (noise free)	AICc	CPU time (hh:mm:ss)	Number of weights
[5 5]	1.85	2.47	2.63	2,330	00:14:20	315
[7 7]	1.48	2.00	1.94	2090	00:13:30	445
[10 10]	1.34	1.84	1.56	2,510	00:17:15	655
[5 5 5]	2.00	4.43	4.35	2,610	00:19:43	345
[7 7 7]	1.50	2.13	2.35	2,300	00:15:18	501
**[10 10 10]**	**0.982**	**1.05**	**0.54**	**1800**	**00:19:42**	**765**
[15 15]	1.33	1.72	1.62	3,970	00:17:32	1,045
[20 20]	0.922	1.27	1.01	4,250	00:22:51	1,485
[20 20 20]	0.972	1.27	0.98	6,860	00:24:47	1905

The bold values indicate the optimal model configuration.

Comparing the shallow hybrid model with 12 hidden nodes (Table 2) with the deep hybrid model with 3 hidden layers (10 × 10 × 10) (Table 3) shows that the latter has significantly better training and testing metrics. The 3 hidden layers did not correspond to a large increase in the number of weights (only 17.9%). However, the training error decreased 36.2% and more importantly the noise free test error decreased 73.8%. Both the AICc score as the test WMSE point to the hybrid deep structure (10 × 10 × 10) as being the best model. As for the CPU time, despite the higher complexity of the deep model (with 17.9% more parameters), the CPU time was reduced by 20.8%. This is mainly explained by the fact that ADAM with stochastic regularization is practically insensitive to weights initialization requiring a single training event compared to the 10 training repetitions in the case of LMM with cross-validation.

**FIGURE 2:**
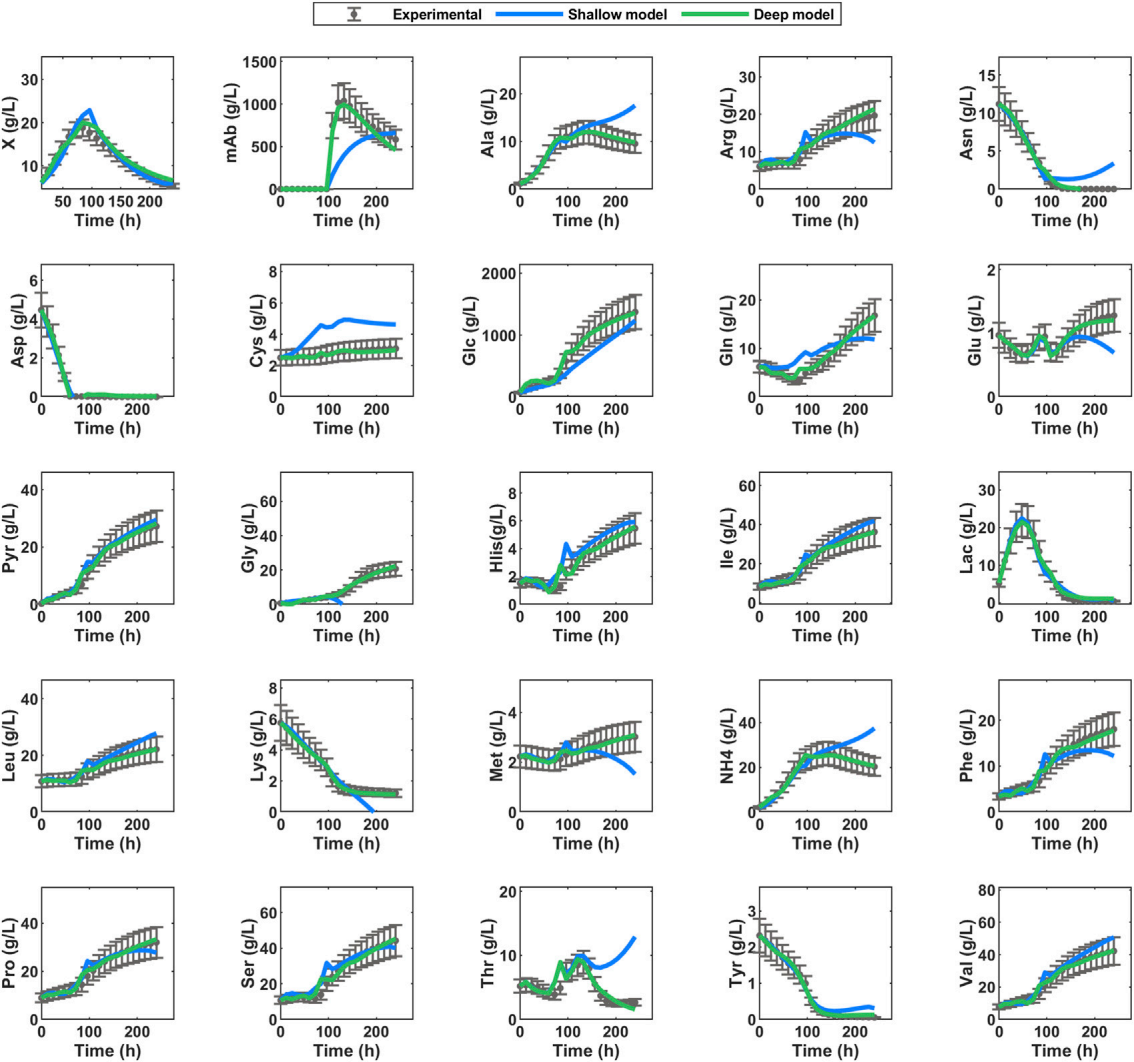
Dynamic simulation of the best shallow (12 hidden modes + *tanh*) and best deep (10 × 10 × 10 + *ReLU*) hybrid models for a test reactor experiment of the CHO-K1 synthetic dataset. Circles are simulated data points and error bars are standard deviations. Green line is the best deep hybrid model structure (10 × 10 × 10) (Table 3); Blue line is the best shallow structure with 12 hidden nodes (Table 2).

Figure 2 shows the prediction of the dynamics in a test experiment by the best shallow and best deep hybrid models. This example shows qualitatively that the deep hybrid model succeeded to predict very faithfully the dynamics of each variable individually (the predicted time profiles of process variables are always within the error bars). Conversely, the shallow hybrid structure shows systematic deviations in different process phases for different variables. As examples, mAb, Ala, Cys, Gly, Asn, Glu, and Thr show systematic deviations in relation to the true profiles.

#### 3.2.1 Comparison between training methods

**FIGURE 3:**
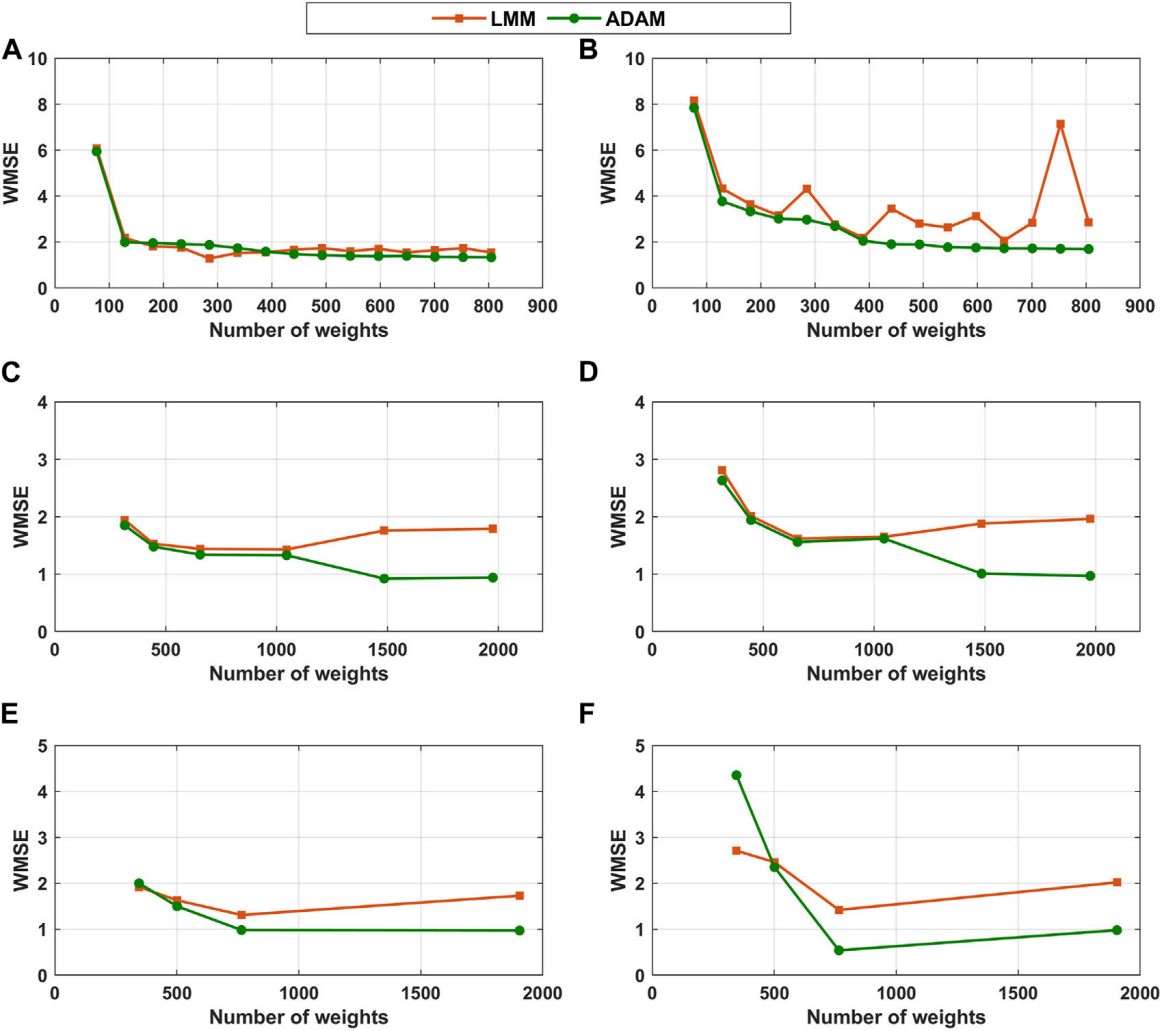
Hybrid model final training and testing errors as function of the FFNN depth (number of hidden layer) and size (number of weights). Orange line and orange squares—hybrid models trained with LMM + indirect sensitivity equations + cross-validation. Green line and green circles—hybrid models trained with ADAM + semidirect sensitivity equations + stochastic regularization. **(A)** Training WMSE of shallow hybrid models (Table 2). **(B)** Testing WMSE of shallow hybrid models (Table 2). **(C)** Training WMSE of hybrid models with 2 hidden layers (Table 3). **(D)** Testing WMSE of hybrid models with 2 hidden layers (Table 3). **(E)** Training WMSE of hybrid models with 3 hidden layers (Table 3). **(F)** Testing WMSE of hybrid models with 3 hidden layers (Table 3).

In order to better understand if the differences if the models performances are due to the training method or to the depth of the FFNNs, the shallow hybrid structures of Table 2 were also trained with the deep learning method (ADAM + semidirect sensitivity + stochastic regularization) and the deep structures of Table 3 were also trained with the classical method (LMM + Indirect sensitivity + cross-validation). The results are shown in Figure 3. Figure 3A shows that the final training error is comparable for both methodologies in the case of shallow hybrid models. The testing error tends to be slightly lower and more stable for shallow hybrid models trained with ADAM. The LMM delivers in some cases equally performing models but it is more unstable. For deep hybrid models with 2 (Figures 3C–F) hidden layers, the differences between both methods are more substantial. For deep structures, as the model size increases the training and testing errors of the ADAM method are significantly lower than those of the LMM method. For large models (number of weights approaching 2000), the difference between ADAM and LMM final training and/or testing errors is as high as 100%. Contrary to ADAM, the final training error delivered by LMM tends to increase with the number of weights suggesting that this approach is unable to exploit the descriptive power of deep FFNNs. However, for small FFNN structures the LMM performs equally or better than the ADAM method.

### 3.3 Hybrid deep modeling of the CHO-K1 fed-batch process

The hybrid modeling framework was applied to the 24 fed-batch experiments collected in a process development campaign to produce a therapeutic glycoprotein. Deep hybrid structures with 2 or 3 hidden layers with nodes between 3 and 30 were investigated. For comparability, single hidden layer hybrid models with 1–18 nodes were also investigated. Given the results of the previous section, only the deep learning method based on ADAM, semidirect sensitivity equations and stochastic regularization was adopted. The training hyperparameters were kept the same as in the synthetic dataset study. The training partition was composed in this case of 20 experiments with 7,953 training examples (83% of data). The testing partition was composed of 4 batches with 1,593 testing examples (17% of data). The training was repeated 10 times for each hybrid model structure with random permutations of test/train experiments to avoid data selection bias, with the results analyzed statistically (resampling method). The 10 train/test permutations were kept the same in all tests performed to ensure comparability. The overall results are shown in Table 4. Structures with less than 8 hidden nodes did not have sufficient complexity to describe the process, showing a very high and unstable training error. The hybrid deep structure (25 × 25 × 25) with 2,855 parameters showed the lowest test error of 1.88 ± 0.44, although 39.3% higher than the training error (1.35 ± 0.21). The best shallow structure with 17 hidden nodes had 16.3% higher training error and more importantly 30.8% higher test error compared to the best deep structure. As in the previous sections, increasing the depth of the FFNN seems to be advantageous in terms of predictive power. The lowest AICc was obtained with the structure (25 × 25 × 25) which also had the lowest test error.

**TABLE 4:** Hybrid modeling results on the experimental CHO-K1 dataset with 24 independent fed-batch experiments and 31 state variables. The activation function in the hidden layers was the *ReLU* in all cases. Hybrid models were trained with ADAM ( α=0.001 , β1=0.9, β2=0.999 and $\eta =1e^{-7}$ ), semidirect sensitivity equations and stochastic regularization (minibatch size = 0.8 and weights dropout = 0.2). For each structure, the training was repeated 10 times with random train/test experiment permutations. Error metrics (WMSE-train, WMSE-test and AICc) are displayed as the mean ± SD of the 10 repetitions.

Number of hidden nodes	WMSE-train	WMSE-test	AICc	CPU time (hh:mm:ss)	Number of weights
7	Unstable	Unstable	Unstable	Unstable	457
8	25.9 ± 0.74	33.6 ± 1.14	70,000 ± 220	01:32:00	518
9	7.39 ± 0.65	9.18 ± 0.89	24,000 ± 150	01:37:00	579
10	3.54 ± 0.40	4.12 ± 0.75	9,075 ± 120	01:40:00	640
11	3.11 ± 0.36	4.09 ± 0.41	6,980 ± 80	02:05:00	701
12	2.61 ± 0.28	3.84 ± 0.62	4,650 ± 60	01:52:00	762
13	1.74 ± 0.29	2.88 ± 0.62	3,920 ± 70	02:01:00	823
14	1.68 ± 0.27	2.74 ± 0.55	3,880 ± 75	02:10:00	884
15	1.60 ± 0.28	2.66 ± 0.54	3,790 ± 60	02:15:00	945
16	1.58 ± 0.28	2.51 ± 0.50	3,775 ± 80	02:17:00	1,006
17	1.57 ± 0.27	2.46 ± 0.42	3,800 ± 70	02:21:00	1,067
18	1.58 ± 0.27	2.47 ± 0.45	4,025 ± 70	02:18:00	1,128
[5 5]	Unstable	Unstable	Unstable	Unstable	365
[7 7]	17.4 ± 0.61	26.9 ± 0.76	50,000 ± 200	01:22:00	513
[10 10]	1.57 ± 0.25	2.50 ± 0.77	3,950 ± 75	01:38:00	750
[5 5 5]	Unstable	Unstable	Unstable	Unstable	395
[7 7 7]	4.61 ± 0.31	5.61 ± 0.69	14,010 ± 100	01:16:00	569
[10 10 10]	1.41 ± 0.21	2.17 ± 0.55	3,750 ± 61	02:13:00	860
[15 15]	1.45 ± 0.22	2.33 ± 0.45	3,890 ± 70	02:21:00	1,185
[20 20]	1.39 ± 0.25	2.10 ± 0.51	3,730 ± 80	02:33:00	1,670
[25 25]	1.38 ± 0.21	2.03 ± 0.49	3,725 ± 60	02:49:00	2,205
[30 30]	1.34 ± 0.23	1.98 ± 0.43	3,630 ± 70	02:59:00	2,790
[20 20 20]	1.37 ± 0.22	2.00 ± 0.48	3,680 ± 70	02:41:00	2090
**[25 25 25]**	**1.35 ± 0.21**	**1.88 ± 0.44**	**3,625 ± 60**	**03:05:30**	**2,855**
[30 30 30]	1.34 ± 0.23	1.95 ± 0.42	3,715 ± 80	03:43:00	3,720

The bold values indicate the optimal model configuration.

### 3.4 Predictive power analysis of the hybrid deep structure (25 × 25 × 25)

**FIGURE 4:**
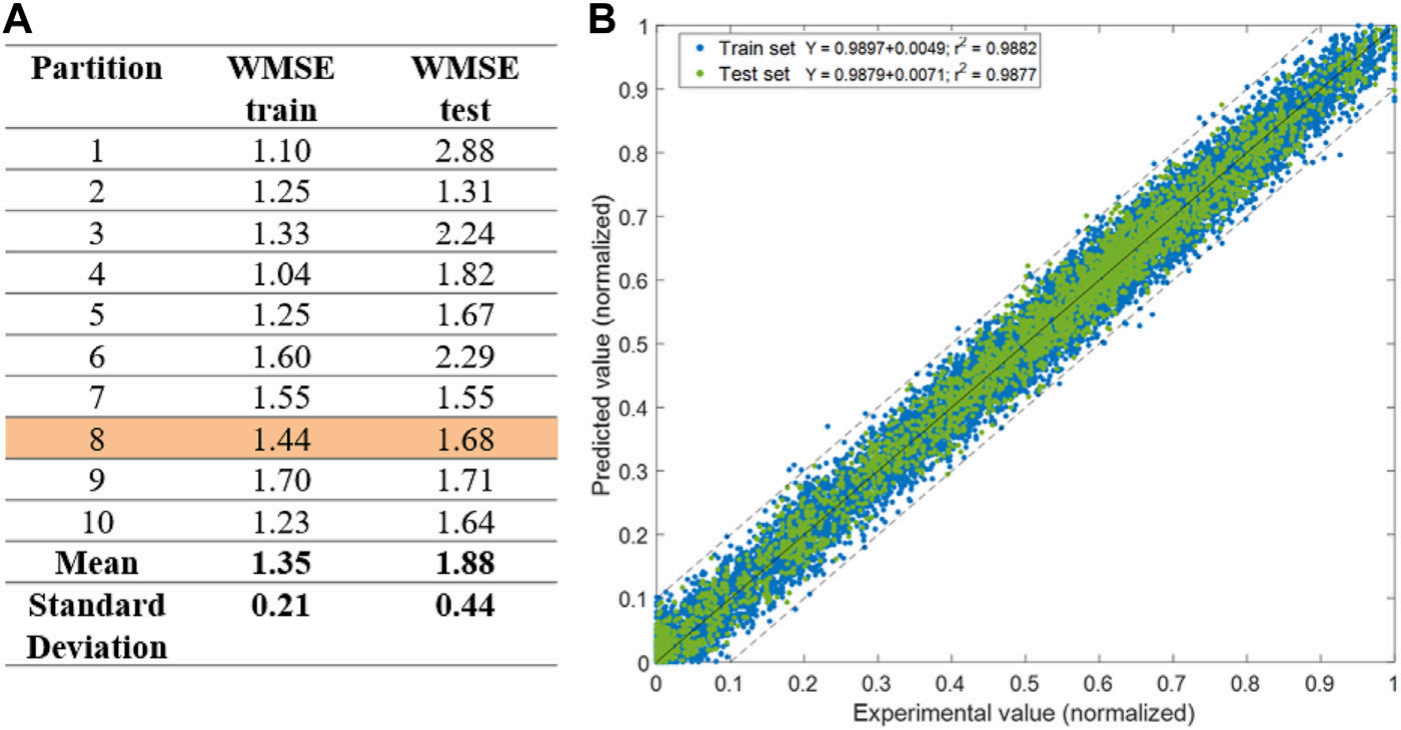
Training results for the best hybrid model structure (25 × 25 × 25) with 2,855 weights. **(A)** Final training and testing error for 10 randomly selected train (20)/test (4) permutations of experiments. **(B)** Predicted over measured concentrations of all biochemical species for training/test partition 8 [highlighted in **(A)**]. Blue circles are training data. Green circles are test data. Full line is the linear regression. Dashed lines are the upper and lower intervals corresponding to one standard deviation. The *r2* is the Pearson correlation coefficient.

The best deep hybrid structure (25 × 25 × 25) was analyzed in more detail. Figure 4A shows the training and test errors obtained for the 10 train/test permutations. The partitioning of data for training and testing has indeed a significant effect on the modeling error metrics. Partition 1 produced a low training error but also the highest test error. Partition 2 produced the best results with both low training and testing errors, and closely matching each other. These results show that the process information content is not equally distributed among the 10 randomly selected train/partitions. This problem can be mitigated with more data added to both the train and test partition in the future. Figure 4B further details model predictions of all concentrations over the respective experimental values for partition 8, which had the closest train and test error to the respective mean values. The slope of the linear regression as well as the Pearson correlation coefficient (*r2*) of train and test data are similar. This shows that despite the slightly larger WMSE for the test partition, there is no significant bias when compared to the train partition data subset.

**FIGURE 5:**
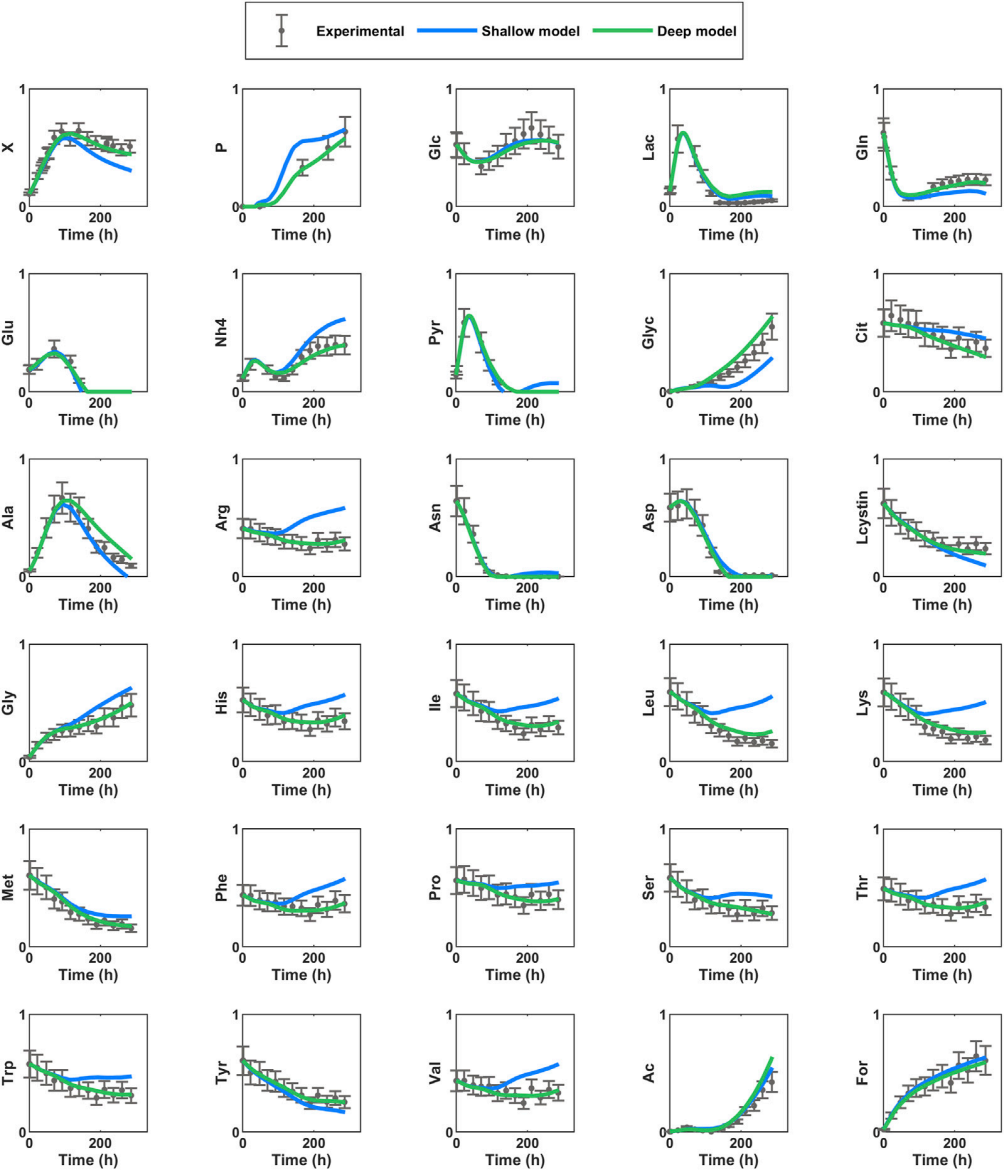
Dynamic simulation of best shallow (17) and best deep hybrid (25 × 25 × 25) models for a test experiment of the CHO-K1 experimental dataset. Circles are experimental data points and error bars are measurement standard deviation. Green line is the best deep hybrid model structure 25 × 25 × 25; Blue line is the best shallow hybrid structure with 17 hidden nodes.

**FIGURE 6:**
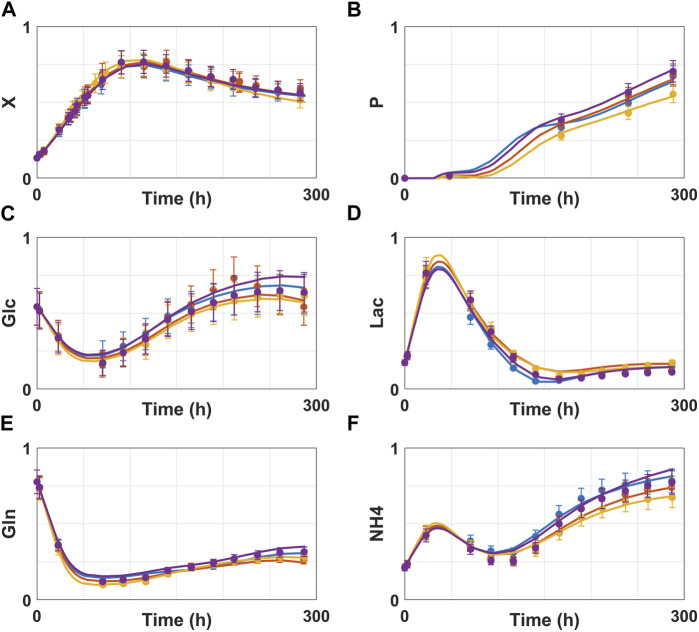
Dynamic simulation of best deep hybrid model (25 × 25 × 25) for multiple test experiments of the CHO-K1 experimental dataset. Circles are experimental data points and error bars are measurement standard deviation. Full lines are model predictions. The color code (symbol + full line) refers to different test experiments of partition 8. Blue, orange, yellow and purple colors represent test experiments 1, 4, 5 and 8 respectively. **(A)** viable cell count. **(B)** glycoprotein titer. **(C)** glucose concentration. **(D)** lactate concentration. **(E)** glutamine concentration. **(F)** ammonium concentration.

The predicted time profiles were analyzed qualitatively for each variable individually. Figure 5 shows the dynamic profiles of the 30 concentrations individually for a selected test experiment (experiment 8) predicted by the best shallow model with 17 hidden nodes and the best deep model (25 × 25 × 25) trained on partition 8. The deep hybrid model follows very closely the measured data. Particularly, viable cells (Xv) and product (P) were accurately predicted. The predictions of metabolites are within the experimental error bars or very close. On the contrary, predictions of the best shallow hybrid model show a tendency to deviate outside of experimental error bounds, especially as the cultivation progresses in time. Figure 6 shows the predicted time profiles for several test experiments for a subset of process variables. It shows that viable cell count, glycoprotein titer, glucose and glutamine concentrations are always predicted within the error bars. Moreover, the switch between lactate production and lactate consumption as well as from ammonium production and ammonium consumption were correctly described by the model.

## 4 Discussion

Hybrid modeling combining First-Principles with neural networks is a well-established methodology in process systems engineering since the early 90s (e.g., von Stosch et al., 2014; Agharafeie et al., 2023). Only very recently hybrid modeling is incorporating deep neural networks and deep learning into its framework (Bangi and Kwon, 2020; Pinto et al., 2022; Bangi and Kwon, 2023). Most hybrid modeling studies of CHO cells followed the shallow approach. The primary goal of this study was to investigate if hybrid deep modeling is advantageous over shallow hybrid modeling in a CHO-K1 process development context.

### 4.1 Is deep hybrid modeling advantageous?

In the case of the synthetic dataset the best shallow model had (12) hidden nodes (Table 2) whereas the best deep structure had 3 hidden layers (10 × 10 × 10) (Table 3). The deep model complexity, as measured by the number of weights, increased only 17.9% in relation to the shallow model. The deep structure achieved a reduction of 36.2% in the training error (WMSE-train), 48.5% in the test error (WMSE-test noisy) and 73.8% in the noise free test error (WMSE-test noise free). All error metrics were significantly improved with emphasis on the noise-free test error, which clearly shows that the deep structure captured more faithfully the intrinsic process dynamics. The CPU time was also reduced by 20.8%. It is noteworthy to mention that the Robitaille et al. (2015) model used to generate the synthetic dataset included the intracellular dynamics of 21 molecular species. The cells accumulated different amounts of intracellular species depending on the reactor feeding conditions eventually triggering different regulatory mechanisms. The deep FFNN is of static nature thus a structural bias could be anticipated due to the mismatch between the dynamic nature of the true process and the structure of the hybrid model. This was however successfully mitigated as reflected in the extremely low noise free test error of extracellular concentrations (Table 3; Figure 2).

In the case of the experimental dataset the best shallow model had 17 hidden nodes whereas the best deep structure had 3 hidden layers (25 × 25 × 25) (Table 4). The model complexity (number of weights) increased in this case quite substantially by 167.6%. The deep structure achieved a reduction of 14.0% in the training error (WMSE-train) and 23.6% in the teste error (WMSE-test) on average. In this case it is impossible to evaluate the noise-free test error reduction. Although the magnitude of the improvement is lower than in the synthetic dataset, it is statistically significant. Moreover, the improvement in the test error is on average higher than in the training error. The training CPU time increased in this case by 31.6%. This increase is explained by the higher model complexity (more 167.6% weights). It becomes clear that CPU time increase does not scale linearly with model complexity (number of weights). This is related with the computation of gradients by the semidirect sensitivity equations (Pinto et al., 2022). In this approach, the sensitivity of state variables in relation to network outputs are independent of the size of the network.

The results obtained for both the synthetic and experimental datasets indicate a clear advantage of deep hybrid models over shallow hybrid models in terms of predictive power. In both cases the test error reduction is significant and always higher than the training error reduction. This suggests that hybrid deep structures capture more faithfully the intrinsic nonlinear dynamics of the true process than the shallow counterpart when exposed to the same training dataset. This eventually translates into more accurate predictions of novel process conditions. This advantage is generally accepted for standalone FFNNs (Goodfellow et al. (2006) and is likely to generalize for hybrid models incorporating deep FFNNs. The only downside to the deep model in this study is the training CPU time increase. Pinto et al. (2022) reported a decrease in prediction error of 18.4% in a Pichia pastoris pilot process using the same training scheme, which is close to the one reported here. In that study, the shallow and deep structures had the same number of weights, and as such the CPU time was also decreased by 43.4%. The CPU cost comparison seems to be case dependent and mainly related with the size of the shallow and deep FFNN embodied in the hybrid model.

### 4.2 What is the best training method?

Two different training methodologies were compared in this study: the classical method and the deep learning method. The classical method is based on the LMM algorithm coupled with indirect sensitivity equations and cross-validation. This method is normally used to train shallow hybrid models (Table 1). The LMM is prone to be trapped in local optima. For this reason, the training must be repeated several times (in our case 10 times) with different parameter initializations for each structure investigated. The deep learning method is based on ADAM, semidirect sensitivity equations and stochastic regularization. ADAM is an improvement of the stochastic gradient descent algorithms with adaptive learning rate. The method estimates the learning rate during the training, based on the first and second moments of the gradients (Kingma, 2014). Only very recently ADAM was applied to train hybrid models (Pinto et al., 2022). A key conclusion was that ADAM is less prone to be trapped in local optima and is practically insensitive to weights initialization. For this reason, the ADAM training was repeated only once for each of the structures investigated, which in theory reduces the CPU time for FFNNs of comparable sizes. Based on the results of Figure 3 with the synthetic dataset, the ADAM method outperforms the classical method based on LMM both in terms of the training and test error especially for deep and large FFNNs. The differences are less marked for shallow and small FFNNs.

### 4.3 What is the optimal network complexity?

Several methods have been proposed to determine the optimal neural network size (Lawrence et al., 1996; Lawrence et al., 1997; Teoh et al., 2006; Mohanan et al., 2022) but there is no consensus on a general methodology. Here, the number of hidden layers and number of nodes in hidden layers were chosen heuristically starting with a single hidden layer with a number of nodes equal to approximately half the number of inputs and then increasing until the optimal size is found. This procedure is replicated with an increasing number of hidden layers. Adding nodes and layers obviously carries a higher number of weights and higher complexity. Thus, choosing the best structure must balance the decrease in error with the increase in model complexity. It is noteworthy to mention that the AICc criterion, which is evaluated on the training dataset only, often fails to discriminate the hybrid structures with the lowest test error. This is an important point because the final hybrid model is expected to faithfully predict unseen process conditions. Unseen process conditions mean that the test data is not yet available. Mei and Smith (2021) have compared probabilistic methods (the AIC and the Bayesian Information Criteria (BIC)) with a resampling method based on blocked cross-validation for selection of shallow FFNNs trained on meteorological data. They concluded that these approaches do not converge to the same conclusions, with the AIC and BIC generally selecting simpler models than the resampling technique. The results in this study show that the AICc and the resampling methods pointed roughly to the same conclusions in the case of hybrid models trained with ADAM (Tables 3 and 4). This means that the lowest AICc score, calculated solely on the training dataset, coincided with the lowest test error statistics produced by the resampling method. Both methods selected the hybrid deep structure (25 × 25 × 25) in the case of the experimental dataset (Table 4) and the hybrid deep structure (10 × 10 × 10) for the case of the synthetic dataset (Table 3). The AICc failed however to discriminate the shallow hybrid model with the lowest test error in the case of the synthetic dataset and the LMM training method (Table 2). It clearly selected a much simpler model in line with the results by Mei and Smith (2021). It is generally accepted that the performance of statistical models should be assessed using resampling methods rather than probabilistic methods (Tashman, 2000). It is thus advisable to apply resampling methods also in the context of hybrid modeling despite the higher CPU cost. In both cases (synthetic and experimental datasets) the optimal depth was 3 hidden layers.

## 5 Conclusion

This study compares for the first time deep and shallow hybrid modeling of a CHO-K1 fed-batch process in a process development campaign. Data of a CHO-K1 cell line expressing a target glycoprotein comprising 24 independent fed-batch experiments with 30 measured state variables were used to compare both methodologies. The results point to a systematic generalization improvement of deep hybrid models with FFNNs with 3 hidden layers over shallow hybrid models. The overall improvement was 14.0% in the training error and 23.6% in the testing error. The CPU time to train the deep hybrid model increased by 31.6% and is mainly related to the higher FFNN complexity. It is today generally accepted that deep neural networks have a general advantage over their shallow counterparts in terms of descriptive power and generalization capacity. This study points to a similar conclusion in a hybrid modeling context. Particularly, deep hybrid models tend to generalize better than shallow hybrid models provided that efficient deep learning algorithms (such as ADAM with stochastic regularization) are adapted to the hybrid model framework. This study focused on FFNN hybrid structures. The combination of first Principles equations with more complex deep neural network architectures, such as convolution neural networks (CNN) and long short-term memory (LSTM) networks, are future research directions in the hybrid modeling field. Shallow hybrid modeling is currently a method of choice in the digitalization of biopharma processes. We expect deep hybrid modeling to further accelerate the deployment of high-fidelity digital twins in the biopharma sector in the near future.

## Data Availability

The datasets presented in this article are not readily available because of industrial intellectual property. Requests to access the datasets should be directed to Rui Oliveira (email: rmo@fct.unl.pt).
