# Supplementary material for: Hybrid deep modeling of a CHO-K1 fed-batch process: combining first-principles with deep neural networks
Source: Front Bioeng Biotechnol. 2023 Sep 8;11:1237963. doi: 10.3389/fbioe.2023.1237963 (PMC10515724; doi:10.3389/fbioe.2023.1237963)
Supplement: Supplementary file 2 [file DataSheet1.docx]

Supplementary Material

Hybrid deep modeling of a CHO-K1 fed-batch: combining First-Principles with deep neural networks

**José Pinto^1^, João R. C. Ramos^1^, Rafael S. Costa^1^, Sergio Rossell^2^, Patrick Dumas^2^, Rui Oliveira^1*^**

^1^ LAQV-REQUIMTE, Department of Chemistry, NOVA School of Science and Technology, NOVA University Lisbon, 2829-516 Caparica, Portugal

^2^ GSK, 89 rue de l'Institut, 1330 Rixensart, Belgium

***Correspondence:**Rui Oliveira
rmo@fct.unl.pt

### Training method of shallow hybrid models

The optimization of network parameters, $w$, is performed over the training data set only in a weighted least-squares sense:

$WMSE=\frac{1}{T} \sum_{t=1}^{T} \frac{{{(c}_{t}^{*}-c_{t})}^{2}}{\sigma_{t}^{2}}$ (S1),

with *T* the number of training examples, $c_{t}^{*}$ the measured concentration at time *t*, $c$ the model predicted concentration at time *t* and $\sigma_{t}$ the measurement standard deviation at time *t*. The Levenberg-Marquardt optimization algorithm (LMM) was employed to minimize the loss function (Eq. S1). The MATLAB function ‘*fminunc*’ with the option of LMM was adopted. The LMM requires the objective function gradients, $g$*.* The gradients equations were obtained by differentiation of Eq. S1 in relation to FFNN parameters, $w$, as follows:

$g=\frac{\partial WSSE}{\partial\omega}=-2\sum_{t=1}^{T} \frac{c_{t}^{*}-c_{t}}{\sigma_{t}^{2}}S_{c,w}$ (S2),

with $S_{c,w}=\left( \frac{\partial c}{\partial w} \right)$ the sensitivity matrix of concentrations to FFFN weights, with ${dim(S}_{c,w})=nc\times nw$. They are called “indirect” because the concentrations are not the FFNN outputs and thus indirectly dependent on the FFNN weights. The indirect sensitivity equations method (Psichogios and Ungar, 1992, Oliveira, 2004) was adopted to compute $S_{c,w}$. Differentiating the hybrid model Eq. 1a in relation to the FFNN weights, $\omega,$ results in the following system of ODEs:

$\frac{dS_{c,w}}{dt}=\left( \frac{\partial f}{\partial c} \right) S_{c,w}+\left( \frac{\partial f}{\partial w} \right)$ (S3a),
$S_{c,w}(t=0)=0$ (S3b).

Eqs. S3a,b were generated automatically using MATLAB’s symbolic differentiation toolbox The integration of the sensitivity equations was performed with a Runge-Kutta 4^th^ order ODEs solver (in-house implementation). At time t=0 the concentrations are independent of FFNN weights and as such the sensitivity values are zero (initial condition S3b). Of note that the system (S3a,b) scales with the number of measured concentrations, $nc$, and number of FFNN weights, $nw$*,* multiplicatively. This may result in very large systems of ODEs with high CPU cost.

### Training method of deep hybrid models

The optimization of network parameters was performed over the training set only in a weighted least-squares sense as for the shallow training method (e.g. by minimizing the cost function value given by Eq. S1). Instead of the LMM, the ADAM method was employed to optimize the network parameters, $\omega$. The ADAM method also requires the gradients $g=\left( \frac{\partial WSSE}{\partial\omega} \right)$but these were computed differently as explained below. A fundamental difference between LMM and ADAM is that the learning rate of each parameter is adjusted based on the first and second moments of the gradients. The detailed equations (Kingma, 2014) are as follows:

$m_{k}= \frac{\beta_{1}\cdot m_{k-1}+(1-\beta_{1})\cdot g_{k}}{(1-\beta_{1}^{k})}$ (S4a),

$v_{k}= \frac{\beta_{2}\cdot v_{k-1}+(1-\beta_{2})\cdot g_{k}^{2}}{(1-\beta_{2}^{k})}$ (S4b),

$w_{k}= w_{k-1}-\frac{\alpha\cdot m_{k}}{(\sqrt{v_{k}}+\varepsilon)}$ (S4c),

with *k* the iteration number, $m_{k}$ the first order moment of gradients, $g_{k}$ the loss function gradients, $v_{k}$ the second order moment of gradients. For the present study, the suggested default hyperparameters of $\alpha=0.001$, $\beta_{1}=0.9$, $\beta_{2}=0.999$ and $\varepsilon={10}^{-8}$ were adopted (Kingma, 2014).

Pinto et al (2022) recently proposed the semidirect sensitivity equations to reduce the CPU cost for the computation of gradients in hybrid deep models. The sensitivity of the loss function to FFNN outputs is computed as follows:

$\left( \frac{\partial WSSE}{\partial r} \right)=-2\sum_{t=1}^{T} \frac{c_{t}^{*}-c_{t}}{\sigma_{i}^{2}}S_{c,r}$ (S5),

with $S_{c,r}=\left( \frac{\partial c}{\partial r} \right)$ the sensitivity matrix of concentration, $c$, to FFNN outputs, $r$, with ${dim(S}_{c,r})=nc\times nr$ . The semidirect sensitivity equations are then obtained by differentiation of the hybrid model Eq. 1a in relation to the FFNN outputs:

$\frac{dS_{c,r}}{dt}=-D S_{c,r}+X$ (S6a),
$S_{c,r}(t=0)=0$ (S6b).

Of note that the system of Eqs. S6a,b is much simpler than the indirect sensitivity Eqs. S3a,b. The key advantage is that the matrix $S_{c,r}$ is much smaller than $S_{c,w}$and independent of the network size. This is particularly interesting for deep and large FFNNs, as the $S_{c,r}$ are independent of the number of hidden layers and number of nodes in the hidden layers. The integration of Eqs. S6a,b was performed with the same Runge-Kutta 4^th^ order ODEs solver (in-house implementation)..

Finally, the loss function gradients $g=\frac{\partial WSSE}{\partial\omega}$ is computed from $\frac{\partial WSSE}{\partial r}$ (Eq. S5) by the well-known error backpropagation algorithm through the network (Werbos, 1974).

$g=\left( \frac{\partial WSSE}{\partial\omega} \right)$=$\left( \frac{\partial WSSE}{\partial r} \right)\left( \frac{\partial r}{\partial w} \right)$ (S7).

Stochastic regularization was employed to avoid overfitting. At each training iteration a subset of training examples (mini-batch) is randomly selected from the uniform distribution. The mini-batch size is defined a priori by the probability (0-1) of a training example being selected at each training iteration. Also, weights-dropout was implemented. At each training iteration, a subset of FFNN weights is dropped out for update in Eqs. S4a-c by random selection from the uniform distribution. The weights dropout probability is defined by a value between 0-1.

**References**

KINGMA, D. P. B., J. 2014. Adam: A method for stochastic optimization. *arxiv***,** 15.

OLIVEIRA, R. 2004. Combining first principles modelling and artificial neural networks: a general framework. *Computers & Chemical Engineering,* 28**,** 755-766.

PINTO, J., MESTRE, M., RAMOS, J., COSTA, R. S., STRIEDNER, G. & OLIVEIRA, R. 2022. A general deep hybrid model for bioreactor systems: Combining first principles with deep neural networks. *Computers & Chemical Engineering,* 165.

PSICHOGIOS, D. C. & UNGAR, L. H. 1992. A Hybrid Neural Network-1st Principles Approach to Process Modeling. *Aiche Journal,* 38**,** 1499-1511.

WERBOS, P. 1974. *Beyond regression new tools for prediction and analysis in behavioral sciences Harvard University.*
